# Oral food challenge unmasks porto-systemic shunting: a case report of a novel provocation test for congenital porto-systemic shunts

**DOI:** 10.1093/ehjcr/ytag601

**Published:** 2026-08-11

**Authors:** Niromila Nadarajan, Alessandro Giardini, Alastair Baker, Michael A Quail

**Affiliations:** Research Department of Children’s Cardiovascular Disease, Institute of Cardiovascular Science, University College London, London WC1N 1DZ, UK; Department of Paediatric Cardiology, Great Ormond Street Hospital for Children NHS Foundation Trust, Great Ormond Street, London WC1N 3JH, UK; Paediatric Liver Centre, King’s College Hospital NHS Foundation Trust, Denmark Hill, London SE5 9RS, UK; Department of Paediatric Cardiology, Great Ormond Street Hospital for Children NHS Foundation Trust, Great Ormond Street, London WC1N 3JH, UK; Paediatric Liver Centre, King’s College Hospital NHS Foundation Trust, Denmark Hill, London SE5 9RS, UK; Research Department of Children’s Cardiovascular Disease, Institute of Cardiovascular Science, University College London, London WC1N 1DZ, UK; Department of Paediatric Cardiology, Great Ormond Street Hospital for Children NHS Foundation Trust, Great Ormond Street, London WC1N 3JH, UK

**Keywords:** Congenital porto-systemic shunt, Total anomalous pulmonary venous connection, Phase-contrast MRI, Oral food challenge, Porto-systemic shunting, Endovascular closure, Case report

## Abstract

**Background:**

Congenital porto-systemic shunts (CPSS) are rare vascular malformations that may present with borderline haemodynamic significance under resting conditions, complicating decisions regarding intervention. Static, fasting-state assessments may underestimate the true physiological impact of these shunts.

**Case summary:**

A 9-year-old male with repaired total anomalous pulmonary venous connection (TAPVC) presented with progressive breathlessness. Cardiac computed tomography (CT) identified a residual anomalous venous connection between the left atrium and portal vein. Baseline phase-contrast magnetic resonance imaging (MRI) demonstrated a trivial left-to-right shunt not meeting conventional criteria for closure. An oral food challenge was performed to exploit the physiological increase in portal venous flow following feeding. Repeat MRI demonstrated reversal of shunt direction with net porto-systemic shunting, accompanied by a rise in serum bile acids above the upper limit of normal. The patient underwent successful percutaneous embolization.

**Discussion:**

This case introduces the oral food challenge as a novel, generalizable provocation test for CPSS, capable of unmasking physiologically significant porto-systemic shunting not apparent under fasting conditions. Dynamic assessment may inform intervention decisions even in the absence of conventional haemodynamic criteria.

Learning points

- Fasting haemodynamic assessment may underestimate the significance of congenital porto-systemic shunts.
- Phase-contrast MRI combined with a food challenge provides a non-invasive method to assess dynamic shunt physiology and support decisions regarding intervention.

## Introduction

Congenital porto-systemic shunts (CPSS) are rare vascular anomalies characterized by abnormal communications between the portal and systemic venous circulations, with an estimated incidence of 1 in 30 000–50 000 live births.^1^ They arise from aberrant remodelling of fetal hepatic and peri-hepatic circulations and are classified as intra-hepatic (which frequently close spontaneously) or extra-hepatic (Abernethy malformations).^2–4^ Clinical manifestations range from asymptomatic presentations to hyperammonaemia, hepatic encephalopathy, liver tumours, and porto-pulmonary hypertension.^5^ While most reported cases represent primary congenital malformations, residual anomalous venous pathways may cause functional porto-systemic shunting. Portal venous blood flow is highly sensitive to feeding state, with post-prandial splanchnic vasodilation markedly increasing portal inflow.^6^ We describe an oral food challenge used to unmask physiologically significant porto-systemic shunting not apparent under fasting conditions.

## Summary figure

**Figure:**
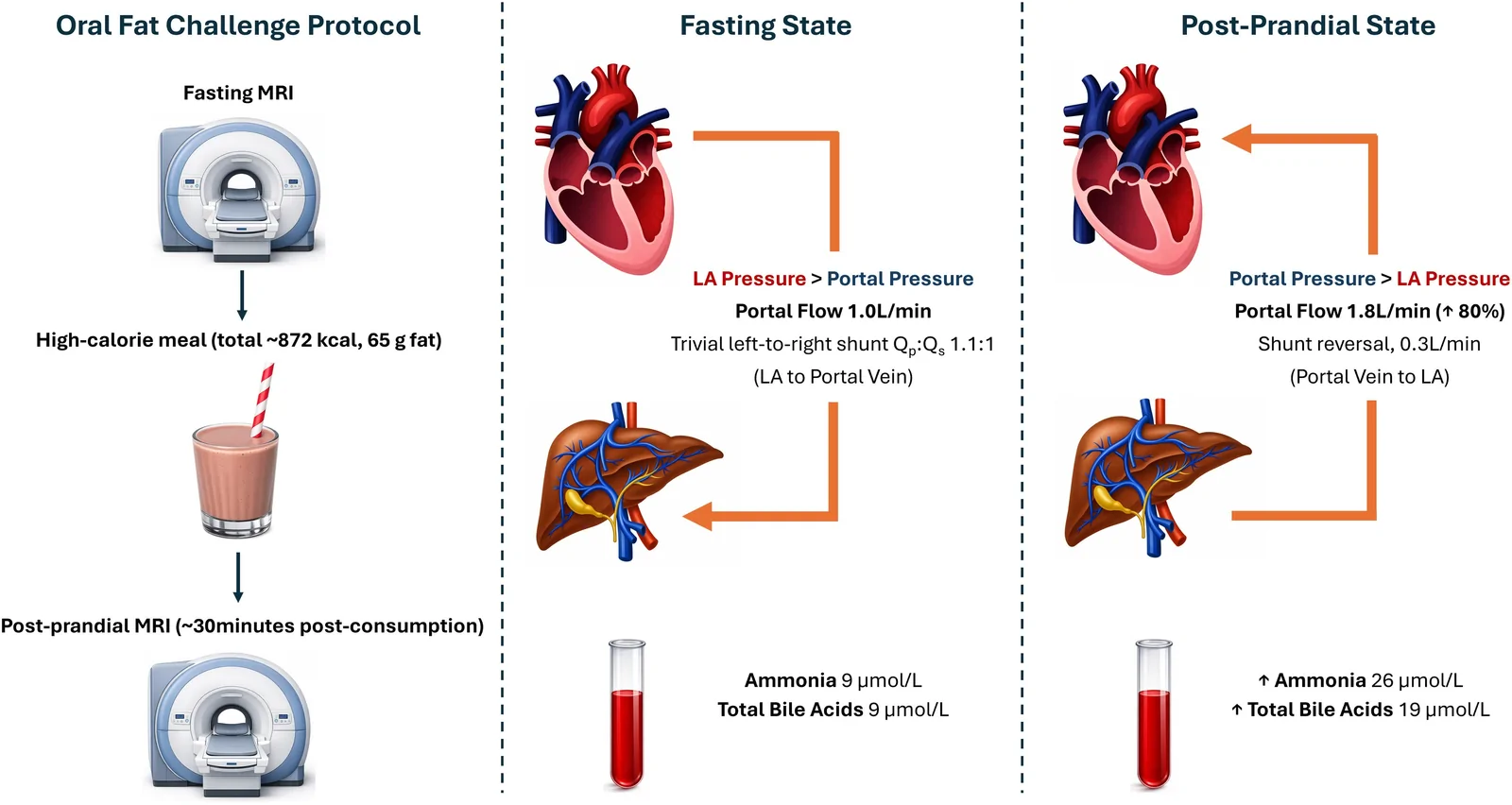


## Case presentation

A 9-year-old male presented with a 1-year history of progressive exertional breathlessness and fatigue. He had undergone surgical repair of TAPVC and reconstruction of the aortic arch at 3 days of life. The original diagnosis was type 2 (cardiac) TAPVC with the venous confluence draining into the coronary sinus, a small atrial septal defect, hypoplastic aortic arch with isthmic coarctation, and a large patent ductus arteriosus. Post-operative course was uncomplicated, with normal growth and development documented until symptom onset at age 8 years. There was no history of hepatic encephalopathy, developmental delay, or neurocognitive symptoms.

**Figure 1:**
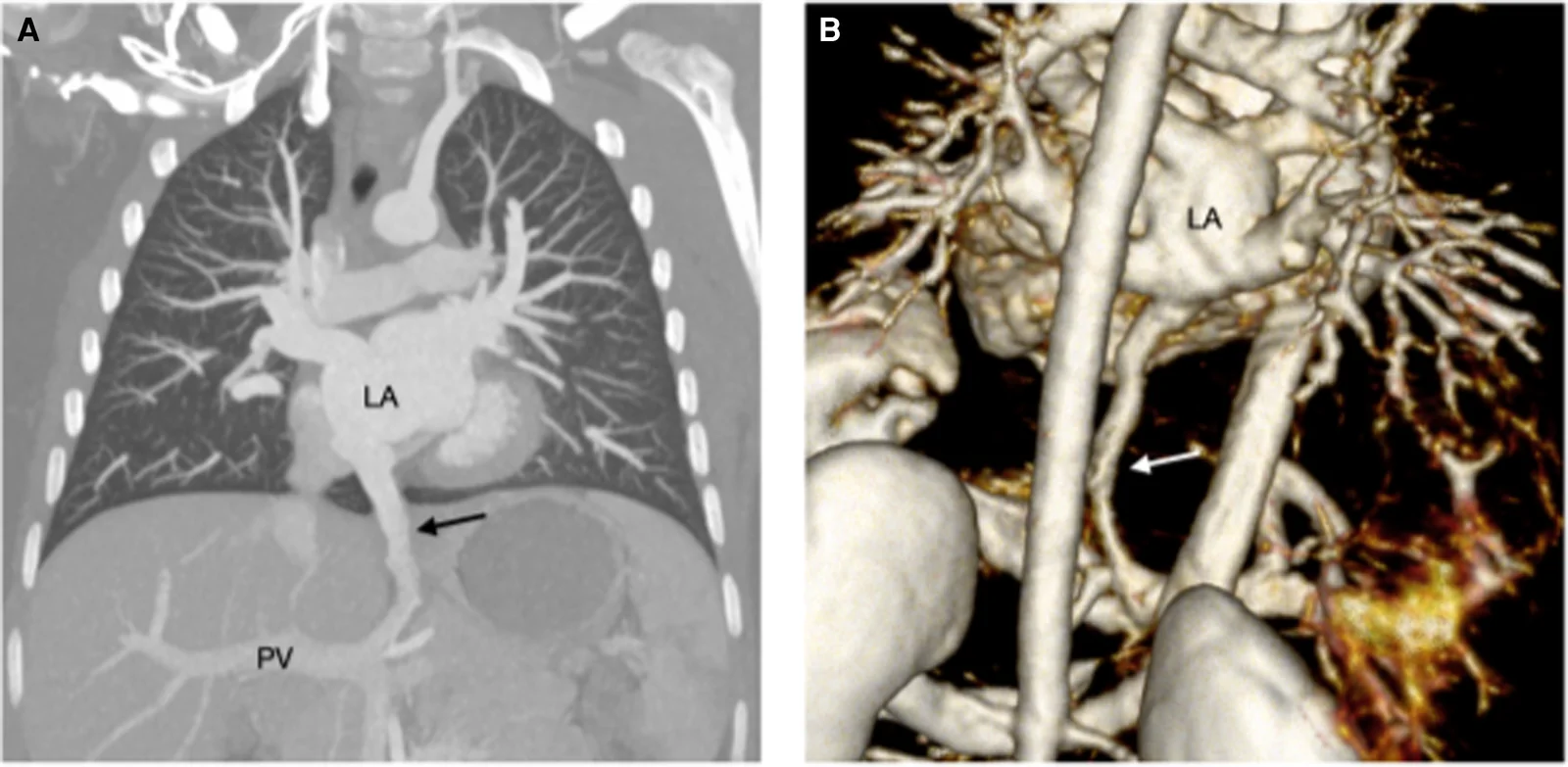
*(A)* ECG-gated cardiac computed tomography (CT) demonstrating the anomalous vertical vein (black arrow) connecting the confluence of the inferior pulmonary veins and left atrium (LA) to the portal vein (PV) at the junction of the superior mesenteric and splenic veins. *(B)* Volume rendered contrast-enhanced magnetic resonance (MR) angiography, posterior projection showing anomalous vertical vein (white arrow).

ECG-gated cardiac CT was performed to evaluate the pulmonary venous anastomosis. This unexpectedly identified a large tubular vertical vein (9 × 9 mm, approximately 5 cm in length) connecting the confluence of the inferior pulmonary veins and left atrium (LA) to the portal vein at the confluence of the superior mesenteric and splenic veins (*Figure 1*). Dedicated hepatic ultrasound demonstrated normal parenchyma, patent portal veins with antegrade flow, and no splenomegaly. Baseline phase-contrast cardiac MRI demonstrated a trivial net left-to-right shunt from the LA to the portal vein of 0.4 L/min, with a Q_p_:Q_s_ of 1.1:1 and normal bi-ventricular volumes and systolic function. Computed tomography and MRI demonstrated no evidence of pulmonary arteriovenous malformation.

The critical clinical question was whether this connection could function as a porto-systemic shunt under physiological conditions. We hypothesized that post-prandial increases in portal flow could transiently raise portal pressure above LA pressure, reversing shunt direction. A two-stage phase-contrast MRI study was designed and performed under fasting and post-prandial conditions.^7^ Serum ammonia and bile acid levels were measured before each stage.

Following an overnight fast, portal vein flow was 1.0 L/min with a trivial net left-to-right shunt of 0.1 L/min (LA to portal vein). After consumption of a high-calorie meal consisting of breakfast (∼480 kcal, 29 g fat) and a standardized milkshake (∼392 kcal, 36 g fat; total ∼872 kcal, 65 g fat), portal vein flow increased by 80% to 1.8 L/min. The direction of flow within the anomalous connection reversed, with net shunting from the portal vein to the LA of 0.3 L/min (*Table 1*). Serum ammonia rose from 9 to 26 µmol/L (within the normal range of 0–40 µmol/L), whilst total bile acids increased from 9 to 19 µmol/L (exceeding the upper limit of normal of 10 µmol/L), consistent with post-prandial porto-systemic shunting.

**Table 1:** Fasting and post-prandial haemodynamic and biochemical parameters

Parameter	Fasting	Post-prandial
Portal vein flow (L/min)	1.0	1.8
Shunt flow (L/min)	0.1 (LA → PV)	0.3 (PV → LA)
Serum ammonia (µmol/L)	9	26
Total bile acids (µmol/L)	9	19^a^

^a^Above upper limit of normal (0–10 µmol/L). LA, left atrium; PV, portal vein

The patient underwent successful percutaneous embolization. Temporary balloon occlusion for 10 min produced no significant change in portal venous pressure (13 mmHg). Definitive closure was achieved with deployment of a 6mm × 50 mm Cook MReye Embolization Coil. At short-term follow-up, the patient reported improved exercise tolerance and resolution of breathlessness.

## Discussion

This case introduces the oral food challenge as a novel physiological provocation test for the assessment of CPSS. A residual anomalous venous connection between the LA and portal vein was identified 8 years after TAPVC repair. Although pulmonary venous drainage to the LA was unobstructed, the anomalous vessel remained in direct communication with the pulmonary venous confluence/LA and demonstrated bidirectional flow. Whether this represents an unrecognized residual component of type 4 (mixed) TAPVC or an unusual CPSS remains uncertain.

This case has important haemodynamic differences from typical CPSS. Most porto-systemic connections communicate between the portal venous system and low-pressure systemic veins. Here, the connection was to the LA, where pressure is typically higher than in the portal vein, resulting in a small left to right shunt in baseline conditions.

Porto-systemic shunting could therefore only occur when portal pressure transiently exceeded LA pressure, which is precisely what occurs following feeding. This observation has broader implications for the assessment of CPSS. Post-prandial increases in portal blood flow are a universal physiological phenomenon, and any porto-systemic connection would be expected to demonstrate increased shunt magnitude in the fed state. Patients with borderline shunt volumes or equivocal metabolic derangements under fasting conditions may harbour clinically significant shunting that is only apparent post-prandially. The oral food challenge combined with phase-contrast MRI may therefore represent a generalizable, non-invasive provocation test applicable across the spectrum of CPSS.

Hepatic encephalopathy is a recognized complication of CPSS, with a reported incidence of 17%–30% in symptomatic patients.^8^ Serum ammonia increased approximately three-fold post-prandially, although it remained within the normal range. In the absence of an agreed threshold for CPSS closure, this rise was not considered an isolated indication for intervention. However, bile acids rose above the upper limit of normal, supporting portal blood bypassing hepatic metabolism. These biochemical findings were interpreted alongside dynamic flow reversal on MRI as evidence of physiologically significant porto-systemic shunting. At the time of assessment, the shunt magnitude was small and insufficient to result in clinically overt hepatic encephalopathy, and the absence of neurological manifestations is likely explained by the predominantly post-prandial nature of the shunting. This pattern of intermittent, meal-triggered shunting may be more common in CPSS than currently recognized.

If portal or hepatic venous pressures were to increase due to a superimposed cause (e.g. liver disease), this could exacerbate porto-systemic shunting and potentially lead to hepatic encephalopathy, hepatopulmonary syndrome, or porto-pulmonary hypertension.^9^ A further consideration is the potential for systemic embolization and stroke, particularly in the setting of portal venous thrombosis or gastrointestinal sepsis (e.g. appendicitis or Crohn’s disease).^10^

Expert consensus supports pre-emptive closure of extra-hepatic CPSS, even in asymptomatic patients, as these communications are unlikely to close spontaneously and carry a risk of severe, potentially irreversible complications, including neurocognitive impairment and chronic porto-systemic encephalopathy. In this case, dynamic porto-systemic shunting and post-prandial biochemical changes further supported closure, despite the absence of overt neurological symptoms or conventional haemodynamic criteria.^11,12^

Treatment strategies currently include surgical ligation, transcatheter closure, and liver transplantation.^13^ Endovascular techniques have become the preferred approach when anatomy is suitable, with reported improvement in clinical symptoms in up to 96% of paediatric patients.^14,15^

In conclusion, this case represents the first reported use of post-prandial physiological provocation to assess an anomalous porto-systemic connection. Dynamic assessment may inform intervention decisions even in the absence of conventional haemodynamic criteria.

## Data Availability

The data underlying this case report will be shared on reasonable request to the corresponding author.

## Lead author biography

Dr Niromila Nadarajan is a Cardiology Higher Specialty Trainee, Clinical Research Fellow and PhD researcher at the Institute of Cardiovascular Science, University College London, UK. Dr Nadarajan’s clinical and research interests are centred around congenital heart disease, with a specialist interest in single-ventricle congenital heart disease and improving outcomes in patients with Fontan circulations. Her current research focuses on Fontan-associated liver disease, aiming to advance understanding of its pathophysiology through interdisciplinary work spanning congenital cardiology, hepatology and imaging.

## Notes

**Consent:** The authors confirm that written informed consent was obtained from the patient’s parent/legal guardian for submission and publication of this case report in accordance with journal policy. Images for research acquired under Research Ethic Committee approved study (HRA 06/Q0508/124).
